# Supplementary material for: Molecular basis for Cdk1‐regulated timing of Mis18 complex assembly and CENP‐A deposition
Source: EMBO Rep. 2017 Apr 4;18(6):894–905. doi: 10.15252/embr.201643564 (PMC5452045; doi:10.15252/embr.201643564)
Supplement: Supplementary file 1 — Expanded View Figures PDF [file EMBR-18-894-s001.pdf]

# Expanded View Figures

**Figure EV1. Mis18BP1 amino acid conservation and Mis18 complex formation with the well conserved Mis18BP1 N-terminal region (amino acids 20–130) *in vivo*.**

A Alignments include *Homo sapiens* (*hs*), *Mus musculus* (*mm*), *Bos taurus* (*bt*), *Gallus gallus* (*gg*), and *Xenopus tropicalis* (*xt*). Black boxes indicate SANTA and SANT domains, respectively. Multiple sequence alignment (conservation score is mapped from red to cyan, where red corresponds to highly conserved and cyan to poorly conserved) was performed with MUSCLE (MUltiple Sequence Comparison by Log-Expectation, EMBL-EBI) [31] and edited with Aline (sequence alignment editor) [32].

B Representative images (left) and quantification (right) for the recruitment of mCherry-Mis18BP1$_{20–130}$ and mCerulean-Mis18β to the alphoid$^{tetO}$ array in HeLa 3-8 cells expressing TetR-eYFP-Mis18α$_{wt}$. Middle bars show median whilst error bars show SEM. Mann–Whitney test vs. TetR-eYFP; $P \leq 0.0001$, $n \geq 63$. Scale bar, 5 μm.

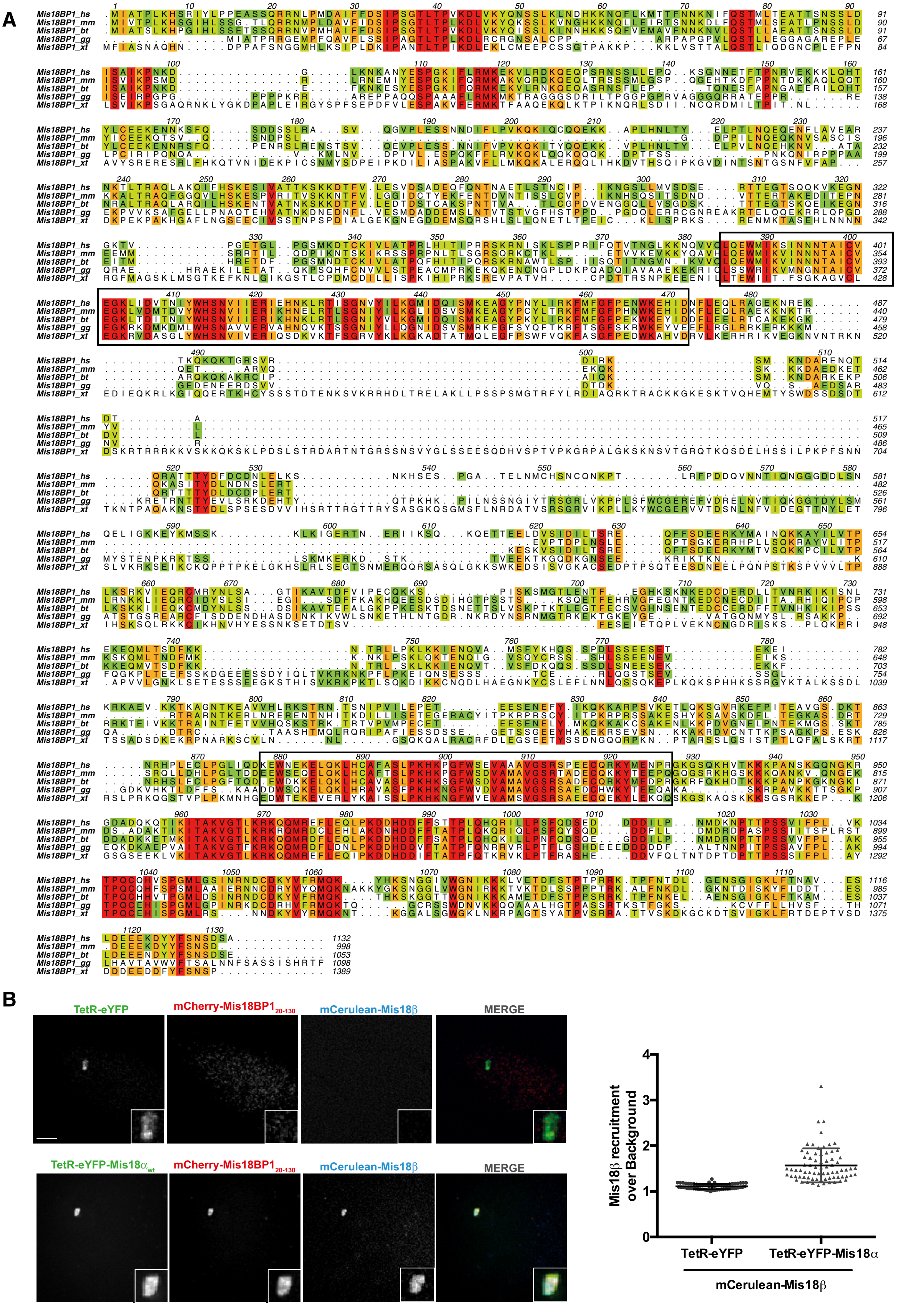


Figure EV1.

 © 2017 The Authors

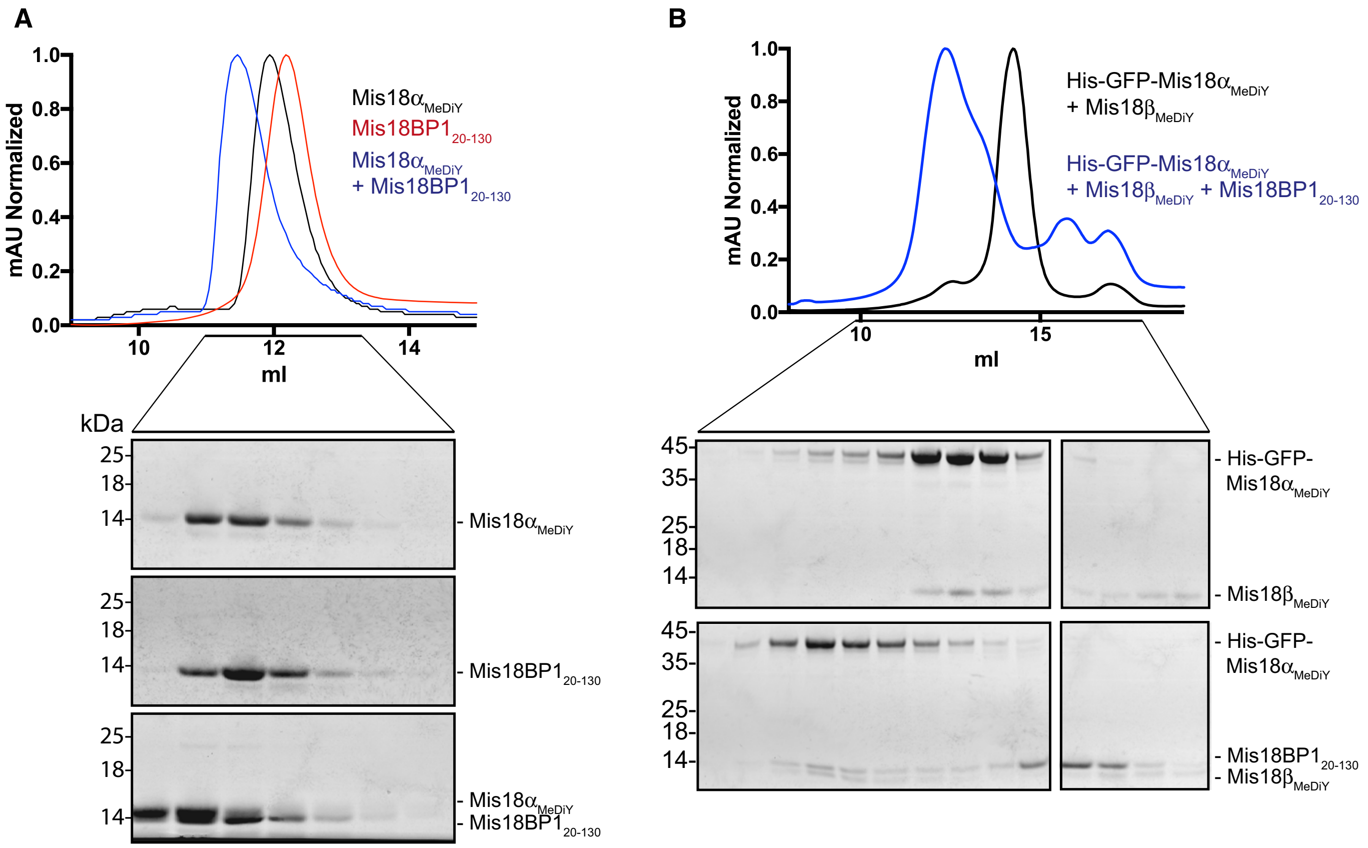


**Figure EV2. Mis18α$_{MeDiY}$ can directly interact with Mis18BP1$_{20–130}$.**

A, B SEC profiles and respective SDS–PAGE analyses of (A) Mis18α$_{MeDiY}$, Mis18BP1$_{20–130}$, and Mis18α$_{MeDiY}$ mixed with two times molar excess of Mis18BP1$_{20–130}$. Mis18α$_{MeDiY}$ elutes at 11.9 ml while Mis18α$_{MeDiY}$/Mis18BP1$_{20–130}$ elutes at 11.5 ml. Proteins were separated using a Superdex 75 10/300 column and (B) His-GFP-Mis18α$_{MeDiY}$/Mis18β$_{MeDiY}$ and His-GFP-Mis18α$_{MeDiY}$/Mis18β$_{MeDiY}$ mixed with two times molar excess of Mis18BP1$_{20–130}$ elutes at 14.3 ml and 12.4 ml, respectively. Proteins were analyzed using a Superdex 200 increase 10/300 column.

© 2017 The Authors

**A**

**Mis18α/β**

| Molecules | calculated MW in kDa |
|---|---|
| 5 | 126.5 |
| 6 | 151.8 |
| 7 | 177.1 |

**B**

**His-GFP-Mis18α/ His-Mis18β**

| Molecules | calculated MW in kDa |
|---|---|
| 3α 3β | 247.5 |
| 4α 2β | 276.0 |
| 5α 1β | 304.5 |

**C**

**His-Mis18α/ His-GFP-Mis18β**

| Molecules | calculated MW in kDa |
|---|---|
| 3α 3β | 247.5 |
| 4α 2β | 221.4 |
| 5α 1β | 195.3 |

**D**

**His-GFP-Mis18$\alpha_{C\text{-}term}$/ His-MBP-Mis18$\beta_{C\text{-}term}$**

| Molecules | calculated MW in kDa |
|---|---|
| 1α 1β | 84.8 |
| 2α 1β | 119.6 |
| 1α 2β | 134.8 |

**E**

**Mis18α/β/ Mis18BP1$_{20\text{-}130}$**

| Molecules (4α 2β + ?) | calculated MW in kDa |
|---|---|
| 1 Mis18BP1 | 165.6 |
| 2 Mis18BP1 | 178.2 |
| 3 Mis18BP1 | 190.8 |

**F**

**Mis18α/β/ His-Sumo-Mis18BP1$_{20\text{-}130}$**

| Molecules (4α 2β + ?) | calculated MW in kDa |
|---|---|
| 1 Mis18BP1 | 179.8 |
| 2 Mis18BP1 | 206.6 |
| 3 Mis18BP1 | 233.4 |

**Figure EV3. Predicted molecular weights for Mis18 complexes with different subunit stoichiometry.**

A–F Predicted MWs of (A) Mis18α/β, (B) His-GFP-Mis18α/His-Mis18β, (C) His-Mis18α/His-GFP-Mis18β, (D) His-GFP-Mis18$\alpha_{C\text{-}term}$/His-MBP-Mis18$\beta_{C\text{-}term}$, (E) Mis18α/β/ Mis18BP1$_{20–130}$, (F) Mis18α/β/His-Sumo-Mis18BP1$_{20–130}$ complexes with different subunit compositions. These values together with the measured MWs from SEC-MALS analysis shown in Fig 3 were used to deduce the correct subunit composition.

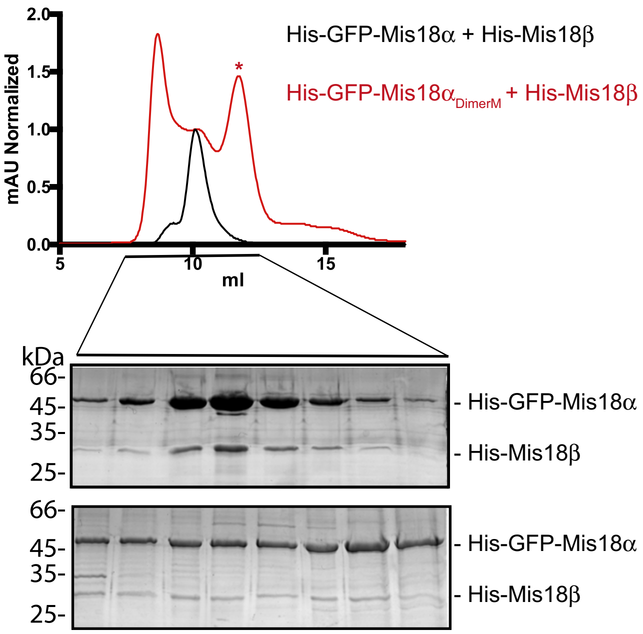


**Figure EV4. Characterization of Mis18$\alpha_{DimerM}$/Mis18β.**

SEC profiles and SDS–PAGE analyses of His-GFP-Mis18α/His-Mis18β and His-GFP-Mis18$\alpha_{DimerM}$/His-Mis18β complexes. SEC analyses were carried out using a Superdex 200 increase 10/300 column. Sample used for SEC-MALS is indicated with an asterisk (*).

 © 2017 The Authors

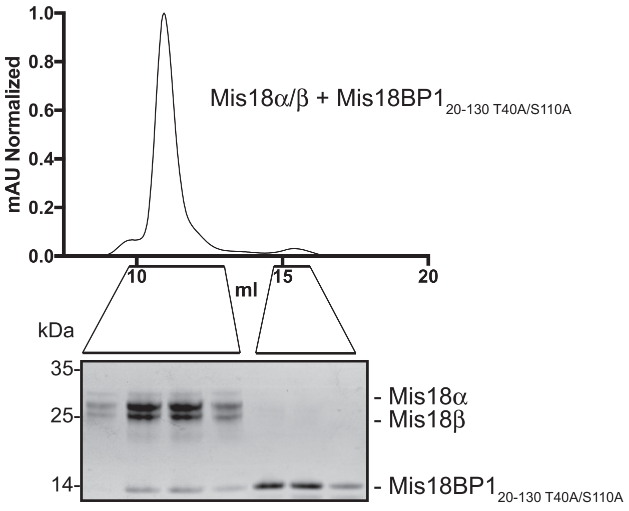


**Figure EV5. Mis18BP1$_{20–130\ T40A/S110A}$ binds Mis18α/β *in vitro*.**
SEC profile and respective SDS–PAGE analysis of Mis18α/β mixed with two times molar excess of Cdk1 non-phosphorylatable mutant, Mis18BP1$_{20–130\ T40A/S110A}$. SEC run was carried out using a Superdex 200 increase 10/300 column.
